# PW02-032 - CNS manifestations and NLRP3/CIAS1 gene mutations

**DOI:** 10.1186/1546-0096-11-S1-A173

**Published:** 2013-11-08

**Authors:** T Kümpfel, E Schuh, R Hohlfeld, P Lohse

**Affiliations:** 1 Institute of Clinical Neuroimmunology, LMU-Munich, München, Germany; 2 Section of molecular genetics, Institute for Laboratory Medicine Blessing, Singen, Germany

## Introduction

Central nervous system (CNS) involvement is common in cryopyrin-associated periodic syndromes (CAPS), especially in children. Neurological manifestations of the low-penetrance cryopyrin mutations V198M and Q703K encoded by exon 3 of the *NLRP3* gene have not been investigated so far.

## Objectives

To determine the frequency of the V198M and Q703K substitutions in adult patients with possible inflammatory CNS disease and at least two symptoms compatible with CAPS and to describe the clinical phenotype of mutation-positive patients.

## Methods

94 unrelated, consecutive patients with possible inflammatory CNS disease and at least two symptoms compatible CAPS were prospectively screened for the V198M and Q703K mutations. In addition, the clinical, laboratory, and MRI features of mutation carriers were assessed.

## Results

15 patients (16%; 12 females) were identified to carry one of the two low-penetrance mutations in exon 3 of the *NLRP3* gene (V198M: n= 2; Q703K: n =13). CAPS-associated systemic symptoms consisted of recurrent inflammation of the eyes, arthralgias, myalgias, urticarial rash, abdominal pain, and severe fatigue. CNS manifestation included optic nerve inflammation and/or atrophy, cranial nerve palsy, migraine, recurrent meningitis, and sensoneurinal hypacusis. Eight patients (53%) fulfilled the diagnostic criteria for multiple sclerosis (MS) according to the McDonald criteria. Brain magnetic resonance imaging (MRI) showed abnormalities in all but one patient.

## Conclusion

So far, the V198M and Q703K mutations have been only rarely described in association with MS or CNS inflammation. We observed a a surprisingly high frequency of these two low-penetrance mutations in the cohort studied, leading to a heterogeneous pattern of CNS manifestations in affected patients. Thus, molecular genetic testing should be considered in patients with an unusual CNS inflammation and/or MS, who report additional symptoms compatible with CAPS.

## Competing interests

T. Kümpfel Grant / Research Support from: TK has received grant support by Novartis Pharma, E. Schuh: None declared, R. Hohlfeld: None declared, P. Lohse: None declared

